# Transcriptomic changes preceding neurodegeneration in a *C. elegans* model of tau and TDP‐43 co‐pathology

**DOI:** 10.1002/alz.087557

**Published:** 2025-01-03

**Authors:** Vaishnavi Jadhav, Randall Eck, Samuel Smukowski, Laura Garcia Toscano, Caitlin S Latimer, Paul N Valdmanis, Brian C. Kraemer, Nicole F Liachko

**Affiliations:** ^1^ University of Washington, Seattle, WA USA; ^2^ VA Puget Sound GRECC, Seattle, WA USA; ^3^ University of Washington, School of Medicine, Seattle, WA USA

## Abstract

**Background:**

Alzheimer’s disease (AD), the most common aging‐associated neurodegenerative dementia disorder, is defined by the presence of amyloid beta (Aβ) and tau aggregates in the brain. However, more than half of patients also exhibit aggregates of the protein TDP‐43 as a secondary pathology. Clinically, AD patients with secondary TDP‐43 pathology have more severe cognitive impairment, more rapid cognitive decline, worse brain atrophy, and a shorter disease course. TDP‐43 is already implicated in neurodegenerative disease as the major pathological protein aggregate in amyotrophic lateral sclerosis (ALS) and frontotemporal lobar degeneration (FTLD‐TDP), two other devastating neurodegenerative diseases. In patients with mixed Aβ, tau and TDP‐43 pathology, TDP‐43 dysfunction may synergize with neurodegenerative processes in AD, worsening disease. Using *C. elegans* models of mixed pathology in AD, we have shown that TDP‐43 specifically synergizes with tau but not Aβ, resulting in enhanced neuronal dysfunction, selective neurodegeneration, and increased accumulation of pathological tau.

**Method:**

To identify cellular responses to mixed tau and TDP‐43, we have identified distinct transcriptomic signatures from two disease‐relevant timepoints preceding frank neuronal loss in *C. elegans*.

**Result:**

We find that early disease associated changes are driven by tau, but a unique gene expression signature characteristic of co‐morbid tau and TDP‐43 emerges during aging. Using gene ontology and differential splicing analyses, we find significant changes in genes and pathways including lysosomal mediated degradation, lipid metabolism. We have characterized novel modifiers of tau and TDP‐43 identified from their responsive gene expression profiles in tau and TDP‐43 co‐expressing *C. elegans*.

**Conclusion:**

Establishing early cellular responses to co‐morbid tau and TDP‐43 is critical for understanding protective and pathogenic responses to mixed proteinopathies, and an important step in developing therapeutic strategies protecting against pathological tau and TDP‐43 in AD.

